# Supplementary material for: Subcutaneous sarilumab for the treatment of hospitalized patients with moderate to severe COVID19 disease: A pragmatic, embedded randomized clinical trial
Source: PLoS One. 2022 Feb 25;17(2):e0263591. doi: 10.1371/journal.pone.0263591 (PMC8880885; doi:10.1371/journal.pone.0263591)
Supplement: S1 Table — (DOCX) [file pone.0263591.s002.docx]

**Supplementary Table 1. Full list of medication administration during COVID hospitalization**

| **Medications** | **Sarilumab**  **(N=20)** | **SOC**  **(N=30)** | **Total**  **(N=50)** |
| --- | --- | --- | --- |
| ACE/ARB | 8 (40.0%) | 8 (26.7%) | 16 (32.0%) |
| Lisinopril | 5 (25.0%) | 8 (26.7%) | 13 (26.0%) |
| Losartan | 2 (10.0%) | 0 (0%) | 2 (4.0%) |
| Valsartan | 1 (5.0%) | 0 (0%) | 1 (2.0%) |
| Antibiotics | 17 (85.0%) | 29 (96.7%) | 46 (92.0%) |
| Amoxicillin | 0 (0%) | 1 (3.3%) | 1 (2.0%) |
| Ampicillin | 1 (5.0%) | 0 (0%) | 1 (2.0%) |
| Azithromycin | 5 (25.0%) | 10 (33.3%) | 15 (30.0%) |
| Cefadroxil | 0 (0%) | 1 (3.3%) | 1 (2.0%) |
| Cefepime | 1 (2.0%) | 4 (13.3%) | 5 (10.0%) |
| Cefoxitin | 0 (0%) | 1 (3.3%) | 1 (2.0%) |
| Ceftaroline | 0 (0%) | 1 (3.3%) | 1 (2.0%) |
| Ceftriaxone | 2 (10.0%) | 10 (33.3%) | 12 (24.0%) |
| Cephalexin | 0 (0%) | 1 (3.3%) | 1 (2.0%) |
| Doxycycline | 0 (0%) | 3 (10.0%) | 3 (6.0%) |
| Ertapenem | 1 (5.0%) | 0 (0%) | 1 (2.0%) |
| Meropenem | 1 (5.0%) | 0 (0%) | 1 (2.0%) |
| Metronidazole | 1 (5.0%) | 1 (3.3%) | 1 (2.0%) |
| Piperacillin-Tazobactam | 1 (5.0%) | 1 (3.3%) | 1 (2.0%) |
| Sulfamethoxazole | 1 (5.0%) | 1 (3.3%) | 1 (2.0%) |
| Vancomycin | 3 (15.0%) | 6 (20.0%) | 9 (18.0%) |
| Anticoagulants | 20 (100.0%) | 30 (100.0%) | 50 (100.0%) |
| Apixaban | 4 (20.0%) | 2 (6.7%) | 6 (12.0%) |
| Enoxaparin | 12 (60.0%) | 20 (66.7%) | 32 (64.0%) |
| Fondaparinux | 1 (5.0%) | 0 (0%) | 1 (2.0%) |
| Heparin | 6 (30.0%) | 7 (23.3%) | 13 (26.0%) |
| Rivaroxaban | 0 (0%) | 1 (3.3%) | 1 (2.0%) |
| Warfarin | 1 (5.0%) | 3 (10.0%) | 4 (8.0%) |
| Glucocorticoids | 17 (85.0%) | 26 (86.7%) | 43 (86.0%) |
| Dexamethasone | 15 (75.0%) | 23 (76.7%) | 38 (76.0%) |
| Hydrocortisone | 2 (10.0%) | 1 (3.3%) | 3 (6.0%) |
| Methylprednisolone | 1 (5.0%) | 1 (3.3%) | 2 (4.0%) |
| Prednisone | 0 (0%) | 2 (6.7%) | 2 (4.0%) |
| Statins | 14 (70.0%) | 19 (63.3%) | 33 (66.0%) |
| Atorvastatin | 9 (45.0%) | 14 (46.7%) | 23 (46.0%) |
| Pravastatin | 1 (5.0%) | 0 (0%) | 1 (2.0%) |
| Rosuvastatin | 3 (15.0%) | 2 (6.7%) | 5 (10.0%) |
| Simvastatin | 1 (5.0%) | 3 (10.0%) | 4 (8.0%) |
| Convalescent plasma | 0 (0%) | 1 (3.3%) | 1 (2.0%) |
| Hydroxychloroquine | 1 (5.0%) | 1 (3.3%) | 2 (4.0%) |
| Remdesivir | 15 (75.0%) | 25 (83.3%) | 40 (80.0%) |
| Rituximab | 0 (0%) | 1 (3.3%) | 1 (2.0%) |
| Tocilizumab | 0 (0%) | 1 (3.3%) | 1 (2.0%) |
|  |  |  |  |
| ACE = angiotensin converting enzyme inhibitor; ARB = angiotensin receptor blocker |  |  |  |
