# Supplementary material for: Subcutaneous sarilumab for the treatment of hospitalized patients with moderate to severe COVID19 disease: A pragmatic, embedded randomized clinical trial
Source: PLoS One. 2022 Feb 25;17(2):e0263591. doi: 10.1371/journal.pone.0263591 (PMC8880885; doi:10.1371/journal.pone.0263591)
Supplement: S2 Table — (DOCX) [file pone.0263591.s003.docx]

**Supplementary Table 2. Symptomatology on admission**

| **Symptoms of interest** | **Sarilumab**  **(N=20)** | **SOC**  **(N=30)** | **Total**  **(N=50)** |
| --- | --- | --- | --- |
| Patients reported symptoms within 30 days prior to COVID admission^0^ | N = 17 | N = 27 | N = 44 |
| Cold | 6 (35.3%) | 13 (48.1%) | 19 (43.2%) |
| Cough | 10 (58.8%) | 15 (55.6%) | 25 (56.8%) |
| Fever | 8 (47.1%) | 19 (70.4%) | 27 (61.4%) |
| Shortness of breath | 9 (52.9%) | 18 (66.7%) | 27 (61.4%) |
| Diarrhea | 4 (23.5%) | 7 (25.9%) | 11 (25%) |
| Headache | 3 (17.6%) | 6 (22.2%) | 9 (20.5%) |
| Abdominal pain | 0 (0%) | 2 (7.4%) | 2 (4.5%) |

^0^ The symptoms were captured with a query combining natural language processing and structured data extraction.
