# Supplementary material for: Subcutaneous sarilumab for the treatment of hospitalized patients with moderate to severe COVID19 disease: A pragmatic, embedded randomized clinical trial
Source: PLoS One. 2022 Feb 25;17(2):e0263591. doi: 10.1371/journal.pone.0263591 (PMC8880885; doi:10.1371/journal.pone.0263591)
Supplement: S3 Table — (DOCX) [file pone.0263591.s004.docx]

**Supplementary Table 3. Laboratory tests ordered between ED arrival and randomization**

| **Median laboratory measure^0^** | **Sarilumab**  **(N=20)** | **SOC**  **(N=30)** | **Total**  **(N=50)** |
| --- | --- | --- | --- |
| Creatinine^1^, mg/dL | 1.2 | 1.2 | 1.2 |
| N, (range) | 20, (0.5 – 1.9) | 30, (0.8 – 5.9) | 50, (0.5 – 5.9) |
| eGFR^1^, mL/min/1.73 m^2^ | 79 | 74.5 | 78 |
| N, (range) | 20, (33 – 162) | 30, (11 – 132) | 50, (11 – 162) |
| Neutrophils^2^, K/cmm | 3.9 | 4.3 | 3.9 |
| N, (range) | 19, (1.5 – 8.0) | 30, (1.8 – 25.0) | 49, (1.5 – 25.0) |
| Lymphocytes^2^, K/cmm | 0.7 | 0.7 | 0.7 |
| N, (range) | 18, (0.3 – 1.7) | 30, (0.2 – 2.9) | 48, (0.2 – 2.9) |
| Platelets^2^, K/cmm | 158 | 171.5 | 167.5 |
| N, (range) | 20, (23 – 305) | 30, (65 – 364) | 50, (23 – 364) |
| ALT^1^, K/cmm | 31.5 | 34 | 34 |
| N, (range) | 18, (14 – 132) | 29, (9 – 85) | 47, (9 – 132) |
| Hemoglobin^2^, g/dL | 11.8 | 12.6 | 12.4 |
| N, (range) | 20, (9.4 – 14.2) | 30, (8.8 – 16.5) | 50, (8.8 – 16.5) |
| Ferritin^1^, ng/mL | 428 | 557 | 527 |
| N, (range) | 15, (123 – 6055) | 24, (92 – 3222) | 39, (92 – 6055) |
| D-dimer^1^, ng/mL | 508 | 404 | 428 |
| N, (range) | 17, (185 – 2036) | 25, (193 – 2122) | 42, (185 – 2122) |
| Troponin-I^1^, ng/mL | 0.1 | 0 | 0 |
| N, (range) | 13, (0 – 0.2) | 19, (0 – 0.3) | 32, (0 – 0.3) |
| CRP^1^, mg/L | 73 | 96 | 91 |
| N, (range) | 16, (7.2 – 198) | 26, (4.0 – 380) | 42, (4.0 – 380) |
| Albumin^2^, g/dL | 3.0 | 3.0 | 3.0 |
| N, (range) | 19, (2.4 – 3.6) | 27, (1.4 – 3.9) | 46, (1.4 – 3.9) |
| Procalcitonin^1^, ng/mL | 0.1 | 0.2 | 0.2 |
| N, (range) | 13, (0 – 0.4) | 20, (0 – 2.6) | 33, (0 – 2.6) |

^0^ The laboratory results were summarized as the most adverse level captured during the measurement period: ^1^ defined as the highest value and ^2^ defined as the lowest value.

ALT = alanine aminotransferase; CRP = C reactive protein; eGFR = estimated glomerular filtration rate.
