# Supplementary material for: Subcutaneous sarilumab for the treatment of hospitalized patients with moderate to severe COVID19 disease: A pragmatic, embedded randomized clinical trial
Source: PLoS One. 2022 Feb 25;17(2):e0263591. doi: 10.1371/journal.pone.0263591 (PMC8880885; doi:10.1371/journal.pone.0263591)
Supplement: S4 Table — (DOCX) [file pone.0263591.s005.docx]

**Supplementary Table 4. Comparison of selected clinical variables between patients who died or survived within 30 days of study period**

| **Risk factor, medication, or biomarker^0^** | **Deceased**  **(N=8)** | **Survived**  **(N=42)** |
| --- | --- | --- |
| Risk factor |  |  |
| Age, (median, range) | 76.7 (70.3 – 98.8) | 73.1 (31.0 – 95.3) |
| Age >75 years | 5 (62.5%) | 16 (38.1%) |
| Age >80 years | 2 (25.0%) | 10 (23.8%) |
| BMI, (median, range) | 35.8 (26.2 – 47.9) | 31.7 (22.6 – 48.5) |
| BMI >30 kg/m^2^ | 6 (75.0%) | 25 (59.5%) |
| BMI >35 kg/m^2^ | 6 (75.0%) | 11 (26.2%) |
| History of diabetes | 6 (75.0%) | 19 (45.2%) |
| History of chronic lung disease | 1 (12.5%) | 20 (47.6%) |
| History of renal disease, stage 3 or 4 | 2 (25.0%) | 7 (16.7%) |
| History of cardiovascular disease | 6 (75.0%) | 29 (69.1%) |
| Medication administration during COVID hospitalization |  |  |
| Dexamethasone only | 0 (0%) | 1 (2.4%) |
| Remdesivir only | 1 (12.5%) | 1 (2.4%) |
| Dexamethasone + Remdesivir | 3 (37.5%) | 31 (73.8%) |
| Laboratory tests taken between ED arrival and randomization |  |  |
| CRP^1^, mg/L, median  N, (range) | 87  7, (7.2 – 127) | 95  35, (4.0 – 380) |
| D-dimer^1^, ng/mL, median  N, (range) | 504  7, (204 – 2036) | 419  35, (185 – 2122) |
| Ferritin^1^, ng/mL, median  N, (range) | 440  7, (123 – 2177) | 545  32, (92 – 6055) |
| Lymphocytes^2^, k/cmm, median  N, (range) | 0.7  7, (0.4 – 1.0) | 0.7  41, (0.2 – 2.9) |

^0^ Determined from the medical record as in Tables 2 and 3 and Supplementary Table 3.

The laboratory results were summarized as the most adverse level captured during the measurement period: ^1^ defined as the highest value and ^2^ defined as the lowest value.
