# Supplementary material for: Subcutaneous sarilumab for the treatment of hospitalized patients with moderate to severe COVID19 disease: A pragmatic, embedded randomized clinical trial
Source: PLoS One. 2022 Feb 25;17(2):e0263591. doi: 10.1371/journal.pone.0263591 (PMC8880885; doi:10.1371/journal.pone.0263591)
Supplement: S5 Table — (DOCX) [file pone.0263591.s006.docx]

**Supplementary Table 5. Expected adverse events**

| **Expected adverse events^0^** | **Sarilumab**  **(N=20)** | **SOC**  **(N=30)** |
| --- | --- | --- |
| Neutropenia^1^ | 2 (10.0%) | 0 (0%) |
| Thrombocytopenia^2^ | 1 (5.0%) | 0 (0%) |
| Alanine aminotransferase elevation^3^ | 0 (0.0%) | 1 (3.3%) |
| Non-COVID related infection | 4 (20.0%) | 6 (20.0%) |
| Bacterial | 3 (15.0%) | 5 (16.7%) |
| Fungal | 1 (5.0%) | 1 (3.3%) |
| Oral herpes virus | 2 (10.0%) | 0 (0%) |
| Other adverse events | 1 (5.0%) | 3 (10.0%) |
| Unique number of patients experiencing an expected adverse event | 8 (40.0%) | 7 (23.3%) |

^0^ Adjudicated adverse events captured within 30 days from randomization date.

^1^ Defined as neutrophil count <1,000 mcL; ^2^ defined as platelet count <75,000 mcL; ^3^ defined as more than 3-fold of evaluation from baseline value.
