# Supplementary material for: Subcutaneous sarilumab for the treatment of hospitalized patients with moderate to severe COVID19 disease: A pragmatic, embedded randomized clinical trial
Source: PLoS One. 2022 Feb 25;17(2):e0263591. doi: 10.1371/journal.pone.0263591 (PMC8880885; doi:10.1371/journal.pone.0263591)
Supplement: S6 Table — (DOCX) [file pone.0263591.s007.docx]

|  | N | Events | Mean | SD | Median | 95% Lower | 95% Upper | Probability(Ha) | Probability(Ha3) |
| --- | --- | --- | --- | --- | --- | --- | --- | --- | --- |
| Sarilumab | 14 | 5 | 27.59% | 8.16% | 27.06% | 13.22% | 44.87% |  |  |
| SOC | 15 | 1 | 13.33% | 6.11% | 12.50% | 3.89% | 27.35% |  |  |
| Sarilumab - SOC |  |  | 14.25% | 10.20% | 14.19% | -5.68% | 34.44% | 7.89% | 86.79% |

Table 1i: First Interim analysis, 30 subjects

|  | N | Events | Mean | SD | Median | 95% Lower | 95% Upper | Probability(Ha) | Probability(Ha3) |
| --- | --- | --- | --- | --- | --- | --- | --- | --- | --- |
| Sarilumab | 19 | 5 | 23.53% | 7.17% | 23.00% | 11.09% | 38.91% |  |  |
| SOC | 24 | 1 | 10.26% | 4.80% | 9.57% | 2.94% | 21.38% |  |  |
| Sarilumab - SOC |  |  | 13.27% | 8.63% | 13.09% | -3.28% | 30.70% | 5.78% | 88.75% |

Table 2i: Second Interim, 45 subjects

|  | N | Events | Mean | SD | Median | 95% Lower | 95% Upper | Probability(Ha) | Probability(Ha3) |
| --- | --- | --- | --- | --- | --- | --- | --- | --- | --- |
| Sarilumab | 19 | 5 | 23.53% | 7.17% | 23.00% | 11.09% | 38.91% |  |  |
| SOC | 30 | 1 | 8.89% | 4.20% | 8.27% | 2.53% | 18.66% |  |  |
| Sarilumab - SOC |  |  | 14.64% | 8.31% | 14.39% | -1.04% | 31.66% | 3.36% | 92.56% |

Table 3i: Third interim, requested by DSMB

|  | N | Events | Mean | SD | Median | 95% Lower | 95% Upper | Probability(Ha) | Probability(Ha3) |
| --- | --- | --- | --- | --- | --- | --- | --- | --- | --- |
| Sarilumab | 15 | 2 | 16.67% | 6.69% | 15.91% | 5.85% | 31.66% |  |  |
| SOC | 25 | 0 | 7.50% | 4.11% | 6.78% | 1.62% | 17.32% |  |  |
| Sarilumab - SOC |  |  | 9.16% | 7.84% | 8.78% | -5.42% | 25.61% | 11.15% | 78.64% |

Table 4i: Third interim, requested by DSMB with the first 9 subjects excluded
