# Supplementary material for: Subcutaneous sarilumab for the treatment of hospitalized patients with moderate to severe COVID19 disease: A pragmatic, embedded randomized clinical trial
Source: PLoS One. 2022 Feb 25;17(2):e0263591. doi: 10.1371/journal.pone.0263591 (PMC8880885; doi:10.1371/journal.pone.0263591)
Supplement: S1 File — (PDF) [file pone.0263591.s008.pdf]

Sarilumab for Patients with Moderate COVID-19 Disease: A Randomized Controlled Trial with a Play-The-Winner Design.

Principal Investigator: Westyn Branch-Elliman
Phone: 857-203-5116
E-mail: Westyn.Branch-Elliman@va.gov
Protocol Version Number: 8.0
Protocol Version Date: January 28, 2021

Funding Mechanism: pending
Industry Support provided by: none
ClinicalTrials.gov number: NCT04359901

IND: Exemption Granted by FDA

TABLE OF CONTENTS

1. Protocol Summary/Abstract 3
2. Aims/Objectives 3
3. Background Information 4
4. Rationale and Purpose 5
5. Relevance to Veterans Health 6
6. Study Design 6
7. Study Subject Selection 6
   a. Sample Description 6
   b. Subject Inclusion Criteria 6
   c. Subject Exclusion Criteria 7
   d. Recruitment 7
8. Data Collection/ Study Measures 8
9. Statistical Analysis Plan 16
10. Ethical Issues 21
   a. Risks 21
   b. Potential Benefits 21
   c. Analysis of Risks in Relation to Benefits 22
   d. Stopping Rules 22
11. Safety Monitoring Plan 22
12. Adverse Event/Unanticipated Problems Reporting Plans 27

1. Protocol Summary/Abstract

**Objectives:**	To determine whether blockade of IL-6R is beneficial in patients with COVID-19 infection of moderate severity.
**Research Design:**	Randomized, controlled trial. Two-arm trial comparing standard care alone to standard care with addition of sarilumab (anti-IL6R). The trial will use a randomized play-the-winner design, in which randomization becomes weighted toward the arm that was more effective in previous subjects in the trial.
**Methodology**	Hospitalized patients meeting clinical criteria for moderate disease and testing positive for coronavirus infection. Interventions: sarilumab, 400 mg subcutaneous injection. Standard care is not pre-specified, may vary among patients, and may include agents with anti-viral activity, such as remdesivir or hydroxychloroquine, among others. Up to 120 patients, primary outcome intubation or death within 14 days. Secondary outcomes will include new onset delirium, ICU admission, new onset heart failure, new onset arrhythmia, new or worsening renal failure, and thromboembolic disease. All data will be extracted remotely from the EHR.
**Clinical Implications:**	The study has potential to establish IL-6R blockade, delivered subcutaneously, as standard of care in reducing progression to critical illness in patients with moderate COVID-19 disease.

2. Aims/Objectives

The effectiveness of blockade of IL-6R in treating patients with COVID-19 disease of moderate severity will be tested in a pragmatic and adaptive randomized trial. The primary outcome is intubation or death within 14 days of enrollment OR administration of a rescue dose of IL-6 inhibition if the patient is DNR/DNI and the primary team determines it is appropriate based on clinical deterioration. Secondary outcomes include time to hospital discharge if alive, time to clinical recovery as was defined in a recent study [1], ICU length of stay, time to return to normal or baseline oxygen saturation without subsequent new or increasing supplemental oxygen before hospital discharge, and changes in laboratory biomarkers. Secondary outcomes will include new-onset delirium, ICU admission, new onset heart failure, new onset arrhythmia, new or worsening renal failure, and thromboembolic disease. Secondary clinical outcome status of a subject at will be assessed at time of randomization, time of discharge from hospital, and 30 days post-randomization, utilizing a seven-point ordinal scale [2] as follows[1].

<4	Alive, not hospitalized
4	Hospitalized, no oxygen therapy
5	Hospitalized, oxygen by nasal prongs or mask
6	Hospitalized high-flow O2 or non-invasive ventilation
7	Intubated or ECMO

[1] Note: Points 1-3 of this scale are defined as: 1, not hospitalized and no limitations of activities; 2, not hospitalized, with limitation of activities, home oxygen requirement, or both; 3, hospitalized, not requiring supplemental oxygen and no longer requiring ongoing medical care. As clinical outcome status will be determined utilizing remote data collection from the electronic medical record, it is not feasible to assign statuses corresponding to Points 1-3. Other researchers (Beigel et al) have noted that consideration of Points 1-3 as indicators of a single outcome (defined as “recovery”) is acceptable in the context of COVID-19.

3. Background Information

SARS-COV-2 is a novel human pathogen that emerged at the end of 2019 and rapidly spread worldwide. Clinical disease caused by this novel infection has a high mortality rate, particularly in older adults and in patients with underlying cardiopulmonary disease, populations that are highly prevalent within the VA healthcare system. This creates an urgent need for the VA to quickly and efficiently identify effective therapies to reduce mortality and spread of the disease. Based on in vitro and in vivo data, potential treatment options include anti-viral drugs (such as antimalarials or medications originally developed to treat HIV infection (e.g., lopinavir/ritonavir, remdesivir)), anti-inflammatory drugs, including IL-6 inhibition, and innovations in supportive care, such as placing patients in prone positioning while awake and not intubated. Importantly, in the context of the global pandemic and exponential spread of the disease, clinical trials must be designed in a manner that 1) optimizes outcomes for Veterans, and 2) produces rapid answers, so that effective treatments can be deployed quickly to reduce unnecessary deaths. Based on a review of 384 ongoing clinical trials by the Centre for Evidence-Based Medicine, and a daily search of open clinical trials, many conventional controlled clinical trials of antiviral drugs and cytokine-blocking drugs are in progress and are likely to be completed within a few months; however, it is possible that these trials, which may take months to implement and report results, will not yield any information about how to treat patients until after the peak of the pandemic is over. A secondary challenge is that it is important to identify what works and what does not work quickly, so that manufacturers can understand what medications need to be produced so that supply chains are adequate for treating all patients who might benefit from an intervention. Thus, innovative clinical trial methodologies are needed in order to produce answers in a highly compressed time frame to inform medical decision-making and supply chain management.

This novel and innovative proposal concerns use and comparison an existing drug (sarilumab) that blocks the receptor for the inflammatory cytokine IL-6. Most studies of IL-6 pathway blockade are using IV medications and include restrictive eligibility criteria and are directed towards patients with severe disease. We hypothesize that patients earlier in the disease course – prior to severe respiratory decompensation – might benefit the most from IL-6 inhibition, and thus are the population included in this pragmatic trial. This study will expand our knowledge and understanding about how IL-6 inhibition can be used to prevent disease progression in patients with moderate disease. It will use a more readily available subcutaneous, longer-acting formulation directed towards patients with moderate disease, of whom 25-35% can progress to severe disease, often rapidly. Eligibility criteria allow for a broad range of underlying choices of other medications, including antiviral medications such as remdesivir and hydroxychloroquine, among others, baseline laboratory values, and comorbidities, reflecting likely real-world clinical use. Thus, this pragmatic project would fill a major gap in research about the clinical care of patients with COVID-19.

Release of preliminary results to the popular press already necessitated revision of this protocol after only 9 subjects had been enrolled. Interim analysis of a study of sarilumab by its manufacturer has led to revision of the study population and treatment arms. Originally, two doses (200 mg and 400 mg) were tested, and patients with either severe (oxygen requirement and other poor prognostic features) or critical (mechanical ventilation) disease were included. Interim analysis indicated benefit only with 400 mg in critical disease, so that will be the design of the remainder of that trial. In contrast, a study of a relatively high dose of tocilizumab (8 mg/kg) was reported to be beneficial in a controlled trial of patients varying in severity from critical illness to merely requiring supplementary oxygen. In response, the current study has been modified to allow routine escalation of dosing among a broad range of "moderate" severity short of critical illness. For patients who are critically ill, it is expected that off-label use of a wide range of "heroic" therapies will be tried, including but not limited to cytokine blocking therapies, so exclusion of such patients from this trial does not limit the therapeutic options for such patients, and from a research perspective, such patients have become the focus of the largest ongoing study of sarilumab.

4. Rationale and Purpose

Severe COVID-19 disease is characterized by a severe acute respiratory distress syndrome (ARDS) followed by a severe cardiomyopathy in many cases. Based on currently available data, it is unclear how much of the respiratory damage in COVID-19 is due to direct effects of SARS-COV-2, or if the primary driver of severe disease is the inflammatory response generated in response to the virus. The key role of inflammation in the progression of the disease is highlighted by recent studies, which demonstrate that patients who progress to requiring ICU-level care no longer have detectable virus present in their respiratory tract. Thus, identifying drugs that could dampen the inflammatory response prior to severe clinical decompensation without adversely affecting clearance of virus would be clinically useful and lead to improved outcomes. Elevated biomarkers of severe inflammation (such as IL-6, CRP, ferritin, and lymphopenia) have been strongly associated with severe disease and increased mortality in patients with COVID-19 [3,4], and early development of a strong IgG response may paradoxically be a poor prognostic sign during the acute phase of the disease. In contrast, viral load in upper respiratory secretions at baseline is only modestly associated with clinical outcomes, and viral load declines in most patients after presentation, independent of the clinical course of the disease [5]. Severe disease typically arises more than 2 weeks after symptom onset, a time at which virus is usually no longer detectable. These findings suggest that progression to severe disease may be driven more by the inflammatory response to the disease than a direct viral effect and point to anti-inflammatory drugs as potentially critical therapeutic options. Importantly, high dose glucocorticoids have been found to be ineffective in severe COVID-19 and are associated with somewhat worse outcomes, so other more targeted anti-inflammatory options are urgently needed.

Blockade of IL-6 signaling is a particularly attractive approach. Sarilumab and tocilizumab are antibodies to the IL-6 receptor (IL-6R), FDA-approved for long-term treatment of rheumatoid arthritis. Tocilizumab has been used open-label with enough anecdotal success in enough patients in China [6] that the manufacturers of sarilumab and tocilizumab have rapidly opened clinical trials in multiple countries at assess efficacy in patients with severe COVID-19 disease. Tocilizumab has also demonstrated efficacy in reducing mortality associated with the cytokine release syndrome in patients receiving CAR-T treatment for cancer, in which the pathogenic pathway is widespread T-cell activation, which leads to production of pleiotropic inflammatory cytokines such as IL-6 by monocytes. Although trials attempting to rescue

patients with severe COVID-19 with ARDS and/or cardiomyopathy are essential and are in progress, the ideal population for clinical use may be patients who are not yet critically ill but are at high risk of clinical deterioration due to secondary inflammation.

5. Relevance to Veterans Health

COVID-19 has a high mortality rate of 2-3% among symptomatic patients. It is expected to have a particularly high mortality in Veterans, because age >60, underlying cardiovascular or lung disease, and obesity are significant risk factors for mortality and clinical deterioration. Thus, advances in care of patients sick enough to be hospitalized yet prior to intubation are expected to have particular benefit for Veterans.

6. Study Design

Sites. VA Boston Healthcare System, VA Connecticut Healthcare System, White River Junction VA Medical Center, Providence VA Medical Center, VA Maine Medical Center – Togus.

Design. Prospective, randomized, unblinded interventional clinical trial.

Two arms: standard care, based on established practices within the medical center, or standard care plus subcutaneous sarilumab.

Assignment Strategy: Randomized play-the-winner design, such that randomization becomes weighted toward the arm that was more effective in previous enrolled subjects. The probability of randomization to a specific arm (standard of care or standard of care plus intervention) will be updated based on outcomes in blocks of 15 subjects (see Planned Statistical Analyses). The protocol does not include administration of sarilumab or tocilizumab as a “rescue” medication if a patient randomized to standard of care alone deteriorates to the point that the primary outcome (intubation) is met, or if the patient is DNR/DNI and the patient’s primary clinical team determines intubation would be performed if the patient’s goals of care included intubation. Such patients may receive any treatment per the judgment of their treating physician, and such treatment might include sarilumab or tocilizumab. Use of sarilumab or tocilizumab in this setting will simply be regarded as a treatment failure and not as a protocol deviation.

Data will be extracted remotely from the EHR. No data will be generated specifically for study purposes.

7. Study Subject Selection

a. Sample Description

Study subjects will be inpatients with confirmed SARS-CoV-2 testing. Testing is performed at the discretion of the treating physician. Only Veterans will be enrolled.

b. Subject Inclusion Criteria

1. Positive testing for active infection with novel coronavirus SARS-CoV-2019
2. Patients with moderate COVID-19 disease as defined clinically:

a. Score of 1-3 (out of 3) on a modified Brescia COVID respiratory severity score (BCRSS), elements of which include wheezing or inability to speak complete sentences without effort, respiratory rate ≥22, O2 saturation ≤94% with or without oxygen supplementation, or requiring ≥2L supplemental oxygen to maintain O2 Sat >94% in patients without previously documented hypoxia or baseline oxygenation requirement; either is equal to one point on the score) all within a 24-hour period prior to enrollment, and/or any worsening of chest X-ray (CXR) findings after COVID-19 diagnosis.
b. Worsening of baseline oxygenation by at least 3%, or increase in oxygen requirement by at least 2L, in patients with pre-existing hypoxemia or receiving supplemental oxygen chronically.
c. The BCRSS risk calculation score is available at: https://www.mdcalc.com/brescia-covid-respiratory-severity-scale-bcrss-algorithm

c. Subject Exclusion Criteria

1. Critical disease, defined by need for mechanical ventilation
2. Expected death within 48 hours
3. Patients taking any of the following for chronic inflammatory diseases: glucocorticoids equivalent to prednisone > 10 mg/day (methylprednisolone > 8 mg/day, dexamethasone > 2 mg/day), a JAK inhibitor (tofacitinib, baricitinib, upadacitinib), or a biologic
    a. Use of chronic inhaled steroids is NOT an exclusion
    b. Current or recent short-term use of glucocorticoids for chronic conditions such as COPD or gout is NOT an exclusion.
    c. Current use of glucocorticoids for COVID-19 is NOT an exclusion
    d. Use of biologics for non-inflammatory diseases is NOT an exclusion
4. Receipt of any IL-6 inhibitor within 3 months prior to enrollment in the trial
5. Pregnancy, due to lack of fetal monitoring capabilities
6. Patients enrolled in other interventional clinical trials, including for COVID-19. Patients enrolled in non-interventional studies or receiving non-FDA-approved drugs for compassionate use are not excluded.
7. Patients whose goal of care is comfort measures only
8. Inability to provide informed consent, or absence of a legally authorized representative to provide informed consent.
9. Severe psychiatric disease that prevents compliance with typical medical care.
10. Initial positive test for active infection with novel coronavirus SARS-CoV-2019 was >4 weeks prior to current admission.

d. Recruitment

Recruitment will occur in-person or over the phone in the inpatient areas at participating sites. At VHABHS, this will be at the West Roxbury campus. The possibility of eligible participants will be made by notification of the PI, co-investigators, or study staff by the treating physician and/or by surveillance of any locally developed tracking database available to study investigators, e.g. COVID TRACKER at VHABHS.

Patients identified as likely to have COVID-19 will be approached about participation when they are clinically stable. Study materials delivered to patients may include a plain-language letter explaining

the rationale for the study and what involvement might entail. Potential participants may be approached in person or usually over the telephone to minimize risk of staff exposure to the highly infectious agent, SARS-CoV-2, using approved mechanisms to obtain consent in this way (see Section 8b, Informed Consent Process). Patients who are not currently eligible for inclusion based on clinical stability, but who may become eligible in the near future (e.g., patients with a Brescia score of 0 who may progress to 1-2), will also be consented. Approaching patients at an early time point for informed consent is preferable both for the purposes of recruitment and for ethical reasons, since patients actively in the process of becoming more ill may not be able to make well-informed decisions, and urgent medical care will take precedence. Patients who have indicated interest in enrollment, but initially failed screening will be considered eligible if they subsequently meet criteria even if it would not be possible or appropriate to discuss the study again due to clinical decompensation. Of course, patients can, if able, have the opportunity to ask additional questions or can withdraw. A Legally Authorized Representative (LAR) may alternatively be approached about the study and asked to provide consent.

8. Data Collection/ Study Measures

Study Procedures

a. Screening

- COVID-19 laboratory testing
- Brescia COVID Respiratory Severity Score (1-3 on a scale of 0-3) any time during the 24-hour period prior to enrollment
- Exclusion criteria related to concurrent or recent use of immunosuppressive drugs
- Exclusion based on CMO status, expected death within 48 hours, severe psychiatric disease limiting ability to comply with recommended treatments
- Exclusion criterion of participation in another interventional clinical trial for COVID-19

b. *Informed consent process*. As noted above under Recruitment, patients may be approached about the study and sign the ICF and HIPAA authorization if they meet eligibility criteria, or if it is suspected or known that they are not yet eligible, i.e. if disease is not severe enough for enrollment but too severe for the patient to be discharged. The process of informed consent will most often be performed remotely by a member of the study staff with a witness when the patient is in an inpatient room, usually over the telephone and/or video call via VA Video Connect (VVC) or other VA approved video-based platform (e.g. Facetime, Zoom). A copy of the Informed Consent and HIPAA Authorization will be provided to the patient by the clinical team prior to the call. The process is expected to take 20 minutes and is the only study-related procedure that requires time or attention from the patient. Due to risk of infection for study staff and hospital personnel, it is usually going to be inappropriate to conduct the informed consent process face-to-face, or to collect and store the signed informed consent document. If the patient wishes to participate, they will be asked to confirm their intent and sign the consent and HIPAA forms. Documentation of written informed consent and HIPAA authorization when conducted remotely will be obtained in one of several ways, in compliance with guidelines from the VA Office of Research and Development.

i. Photograph or screen capture using a VA phone or laptop, of the patient's signature on the paper informed consent and HIPAA Authorization and a copy of the informed consent

signed, electronically or manually, by the person obtaining consent and witness with a notation of how consent was obtained and confirmation that the patient signed the consent document. The signed documents will either be printed and stored with other study documents or will be uploaded to a secure server.

ii. Photograph taken by the patient, of his/her signature on the paper informed consent form and HIPAA authorization and sent to the investigator/delegated study team member. The photograph will either be printed and stored with other study documents or will be uploaded to a secure server

iii. DocuSign may be utilized to obtain informed consent and HIPAA authorization from a patient or the patient's LAR. VA Office of Research and Development has approved DocuSign for use in the VA and specifically for use in this study.

Face-to-Face Options for Obtaining Written Informed Consent

As mentioned above direct interaction with the patient by the study team will rarely occur but if there is such an opportunity the following methods are approved to obtain written informed consent:

iv. The person obtaining consent in the presence of the patient and a witness (in the room or on the phone) will review the consent document and address any questions. If the patient wishes to participate, they will be asked to confirm their intent and sign the consent and HIPAA forms. The person obtaining consent and witness will also sign the consent form. A copy of the signed documents will be collected by 1) obtaining a photo of the signed documents or 2) placing the signature page of the ICF in a sealed, clear plastic bag. After leaving the room, the plastic bag will be decontaminated (e.g., with UV light sanitizer, alcohol swab, CAVI wipe, or other antiviral disinfectant wipe). After decontamination, the signature page will be either copied, and a copy of the signature will be maintained in the study files, or the signature page will be scanned into a PDF file and stored in the study folder. Due to the risks of losing the paper form, and the potential for privacy breaches that might arise from misplaced study documents with PHI, after the signature page is copied or scanned, the document will be destroyed (e.g., shredded), and a note will be made in the study file that the original was destroyed and a copy was maintained. This process will maintain staff safety and limit risks of breach of confidentiality for study participants.

v. Use of iMed Consent, an electronic version of the consent created in CPRS includes the same language as is on the approved informed consent form. Use of the iMed consent would require a face-to-face interaction with the patient by the person obtaining consent and witness and then rolling in a computer cart into the patient's room to provide the Epad to sign the consent document electronically. Use of iMed automatically generates a note in CPRS to serve as documentation. While an iMed version of the consent document has been prepared it may not be practical to implement given the current COVID-19 recommended precautions.

Obtaining Informed Consent from a Legally Authorized Representative

If a LAR is providing consent, the consent form and HIPAA Authorization will be emailed to them using Azure RMS encrypted email, and the informed consent process will be conducted by phone or video call using approved VA platforms. The LAR will be asked to print and sign the ICF and either

scan or take a picture of the signature page and send it back to the study team. Alternatively, a screen capture of the LAR's signature will be witnessed using VA-approved technologies such as VVC or Zoom as approved by the ISSO.

In the instance that a LAR does not have access to a printer, the study team will overnight the documents to the LAR with instructions to sign the documents and as above send a photo of the signed ICF and HIPAA forms.

Study staff will enter a standardized note in CPRS that describes the conduct of the informed consent process and how written informed consent was obtained. If eligibility criteria are met, then randomization may proceed as below. If criteria are not met (i.e., BCRSS score = 0), then randomization may be initiated later, when BCRSS > 0) by adding an addendum to this note or an additional note.

c. *Physical examination, laboratory testing and chest imaging* will be performed as indicated clinically and will be directed based on the treating physicians and will not be conducted specifically for research purposes. Exam findings (respiratory effort, wheezing, respiratory rate, oxygen saturation) and chest imaging are included in the BCRSS score but are not beyond standard clinical practice.

d. *Randomization*. Will be performed by a member of the study staff, using an online tool built by the coordinating center, after eligibility criteria have been confirmed as above. First, a dialog box will appear that is only visible to the study staff and is linked to a URL that will perform randomization and fill a box for either "Randomized to usual care" or "Randomized to sarilumab plus usual care". Each of these will generate a note in CPRS. Randomization to "sarilumab plus usual care" generates a prompt and link to order the medication, which can only be done by an investigator. Randomization to "usual care" includes text in the note that the patient may be declared a treatment failure by the treating physicians before the primary outcome (intubation) is met, or if they are DNR/DNI and the primary team feels death is certain otherwise but may be avoidable with treatment. The primary team may choose to order tocilizumab if available in the medical center but is not encouraged to do so as part of this protocol.

   In the event that the randomization application is not functioning, randomization will be performed by the statistician or a designee not involved in the screening or enrollment of subjects, with the result then communicated to the study team.

e. The pharmacy will be notified immediately when the patient is randomized to receive subcutaneous sarilumab and the order has been placed. A copy of the signed consent form will also be sent to the research pharmacist.

f. *Intervention*. Sarilumab 400 mg subcutaneous injection, or no injection. Sarilumab is provided in prefilled syringes/pens containing 200 mg each as is used clinically, and both injections will be given as soon as is convenient after the patient has decided to enroll. Standard of care as directed by the treating clinicians will be offered in both arms.

g. *Clinical follow-up*. The primary and secondary endpoints are all objective and will be extracted from the EHR remotely rather than through interaction with the study subject. All blood draws are done for clinical purposes only. The lab data collected are consistent with standard of care, but since practice may vary among treating physicians, missing lab data will not be considered protocol deviations. No sensitive data will be collected. There are no follow-up study visits or contact with study subjects, but the EHR will be monitored for medical problems for 6 months after surviving patients have been discharged. These data will be considered study data and will not be reported as adverse events.

Clinical data

- Demographics: age in years, sex, race/ethnicity if available in the EHR, body mass index if available in the EHR, smoking status if available in the EHR
- Comorbidities of interest: coronary artery disease, heart failure, cardiac arrhythmia history, COPD, asthma, bronchiectasis, pulmonary fibrosis, diabetes, hypertension
- Date of intubation if applicable
- Date of symptom onset
- Reason the patient was screened for infection with SARS-CoV-2 (e.g., symptoms or high-risk exposure, community-based screening)
- Date of use of rescue IL-6 inhibition in a patient randomized to standard care, if applicable
- Date of death if applicable
- Cause of death if applicable (Note: Cause of death will be manually adjudicated if death occurs within the first four weeks after patient randomization. If death occurs beyond the first four weeks after patient randomization, cause of death will be extracted from the EHR)
- Date of hospital discharge if living
- Date of return to oxygen saturation >94% without supplemental oxygen, or to the patient's baseline if that baseline is <95%
- Date of clinical recovery, defined as body temperature <37.2degC and slight/no cough per patient report, each for 72 hr [1]
- Administration of antibacterial antibiotics, including type, duration, and indication
- Administration of a "rescue" dose of tocilizumab or sarilumab
- Diagnosis/treatment of bacterial pneumonia (at 1 month and 6 months)
- Administration of other medications for COVID-19
- Date(s) of post-hospital discharge visits to VA Emergency Department and reason(s)/indication(s) for visit

Laboratory data

- Results of COVID-19 test for active infection, including date of first positive
- CRP
- Ferritin
- D-dimer
- CBC with differential: WBC, Hgb, platelets, total neutrophil count, total lymphocyte count
- Liver function tests

- Blood chemistry
- Troponin
- CPK
- Procalcitonin
- Microbiology results, including results of sputum cultures, blood cultures, and urine cultures

Imaging data

- CT findings
- CXR findings

h. *Regulatory / Privacy Aspects.* No sensitive data will be collected. No samples are collected for research purposes only. No study data will be released to non-VA investigators unless a de-identified or limited dataset is released later upon publication of findings, as described in the DMAP.

Data Collection

Data collection will be via VA electronic medical records and administrative data. Assessment of outcomes and relevant data elements as well as adverse events is by active or passive collection of data in electronic health records; the study will not attempt to generate any additional tests or procedures. All outcome processing will be conducted by investigators at the Boston CSPCC unaware of treatment group. In this section we describe our approach to ascertainment and assessment of such outcomes.

Data on the primary outcome will also be obtained in parallel by review of the EHR on a daily basis by study staff. Chart review will be necessary, at some point in the study, to accurately determine the timing of the primary endpoint. Doing so during study treatment is essential to determine whether the primary team is considering use of open-label tocilizumab. Because the primary endpoint is objective, there is little or no possibility of bias being introduced by the study staff reviewing charts for this outcome in an unblinded manner.

Site study staff will surveil for Serious Adverse Events for 30 days and record data on an Excel spreadsheet template, located in a site-specific folder with limited access and maintained in a secure server space. Sites will report SAEs to their local IRBs of Record as appropriate/required and will provide the IRB submission forms to the CSPCC via SharePoint as the primary record of this information.

Surveillance for additional safety events between 31 days and six months will be performed by the Coordinating Center using data pulls. These will not be reported as Serious Adverse Events.

As noted above under Recruitment, identifying potentially eligible study subjects will be performed by manual review of the COVID TRACKER database. Thus, advanced methods for identifying outcomes will be focused primarily on potential adverse events during the study and medical complications during the 6 months after the study is completed.

a. *Background*

The Boston MAVERIC Center has 20 years of experience in ascertainment and assessment of outcomes using the VA electronic health record (EHR) data. We will build on this extensive experience to refine a specific procedure for doing the same in this trial in order to accurately identify and assess outcomes. The process involves several clearly defined steps. First, we develop a method to screen the EHR of all participants on a periodic basis for potential cases. This screening consists of comparing diagnosis and procedure codes for enrolled patients to a list of outcome-relevant ICD 10 and CPT codes. Codes that do not match codes on our list will be considered non-events. The development of the screening process and the algorithms for confirming cases follows a systematic approach that we have used at MAVERIC for a number of years. The screening approach is used to identify all potential cases using key elements in the EHR. The screening tool varies for each outcome (i.e. outcome-specific administrative diagnosis codes).

Second, algorithms are developed to collect and analyze data elements that are used to confirm or refute the potential cases (e.g. presence of imaging during a stroke). The algorithms are based on elements of the clinical definitions of the outcomes and are applied to potential cases to determine if the case is confirmed, refuted, or deemed indeterminate. Algorithms for cases are constructed using "gold standard" cases (identified by manual chart review) and data elements obtained from the medical record that are found in the outcome definitions, such as cardiac enzymes for MI. Once the algorithm for a specific case is defined, the algorithm's accuracy is checked by manual review of cases identified by the algorithm.

Third, indeterminate cases undergo manual adjudication via chart review by a trained clinician. We expect to be left with a relatively small number of events that cannot be resolved by the algorithm, but all of these will be referred for adjudication to an outcomes committee. This greatly lessens the workload for manual adjudication. All patients will be followed until death or 6 months after the end of the subject's participation in the study (even if the primary outcome is determined to have occurred) to collect secondary outcomes.

As an example, a pilot study using VA EHR in the Diuretic Comparison Project (CSP #597) was conducted to determine the availability of potential cases and core data elements that would be needed to develop algorithms to assess the primary cardiovascular outcomes and to test this system. A total of 150 medical records were reviewed, 30 in each category for stroke, myocardial infarction and urgent coronary revascularization and 60 records of patients with a diagnosis of acute heart failure. A medical record abstraction form for each outcome was developed based on standard diagnostic definitions that included information on symptom presentation, physical findings, critical laboratory values, radiographic or imaging findings, electrocardiographic results, hemodynamic data and administration of medications and therapeutic interventions.

b. Adjudication of events

In addition to the review of indeterminate cases, we will also manually adjudicate serious adverse events and the secondary outcomes that occur during a subject's participation in the study.

c. Data Collection and Data Sources

Data for this study will be obtained from the medical and administrative data that are collected and maintained by the United States Department of Veterans Affairs (VA) Corporate Data Warehouse (CDW). This database covers the entire veteran population that utilizes the VA and contains individual information on demographic factors, medical history, key laboratory values, procedure codes, and diagnoses (inpatient and outpatient) coded with the ICD-9-CM and ICD-10 classification systems. Healthcare encounters outside of the VA system will not be captured as a part of this project. Data from the various VA databases will be linked together using a unique veteran identification number that is assigned to each veteran at entry into the system.

Vital status will be ascertained, in part, from and cross-referenced with the VA Vital Status File. This file allows for complete ascertainment of death as it pulls data from multiple sources, including: the Beneficiary Identification and Records Locator Subsystem database; the Death Master File from the Social Security Administration; and the National Patient Care Database. Ascertainment of death with this method has demonstrated 98% sensitivity and 98% agreement with the National Death Index. Cause of death will be collected from the National Death Index, as the VA Vital Status File does not aggregate cause of death across the aforementioned data sources.

d. Data Management and Data Security Plans

Boston CSPCC will employ the use of several data management and analysis tools throughout the conduct of the study including Microsoft SQL Server Management Studio and SAS. Additional information on the use of tools for the analysis of study data can be found in the study specific DMAP and Statistical Analysis Plan.

The Boston CSPCC will create and maintain an electronic study database to manage the trial data. All study data will be collected electronically from CPRS and/or CDW and a study-specific web application by the Coordinating Center throughout the duration of the study. There will be limited paper-based study documents.

Study data is housed on secure VA servers, encrypted and protected in accordance with VA policies compliant with FDA requirements, Federal Information Security Management Act and the HIPAA Privacy and Security rules. Data for this study will be stored in two locations: 1) on a VINCI-hosted server and 2) on a MAVERIC-hosted server. Boston CSPCC personnel manage the data access request process for the electronic systems to ensure that data access is appropriate for each individual and the level of the individual access. Study project management will manage the Data Access Request Tracker (DART) activities associated with granting VINCI access to study databases. VA's Office of Information & Technology (OI&T) is responsible for managing other VA system access and ensuring the security and integrity of VA information systems, including the databases and servers housing study data. In accordance with VA Handbooks and Directives, OI&T is responsible for ensuring that appropriate firewalls and data security is implemented and maintained, that data backups are performed and that data may be restored in the event of a system malfunction.

Data security incidents will be reported according to 1058.01. All Boston CSPCC staff will be expected to report data security incidents to the responsible authority immediately after they become aware of the breach. Whenever possible, the reporting of data security incidents will be handled by the Boston CSPCC Associate Center Director for Quality Assurance (ACDQA). This will be done to facilitate communication

between the center and the oversight bodies. In the event that an incident must be reported by a staff member other than the ACDQA, all communication after the initial report will be handled by the Boston CSPCC Center Director, the Boston CSPCC ACDPOC, or the Boston CSPCC ACDQA. [Note: all new and current Boston CSPCC personnel will be trained on reporting data security incidents.]

All local data security incidents will be reported in accordance with VA policy immediately upon discovering the incident to:

1. The Boston Information Security Officer (ISO)
2. The Boston Privacy Officer (PO)
3. The Boston ACOS for Research
4. The Boston CSPCC Quality Assurance department

Data security incidents will be treated as unanticipated problems by the Boston CSPCC and will be reported as such to the VA IRB.

Study data will be coded and stored using a unique study identifier for each participant. Identifiable information will be collected for patient tracking and safety purposes, and kept in an encrypted, password protected file to which a small number of people will have access. Access to the crosswalk file linking the participant's identifiers and their study data will be restricted to the approved personnel at the CSP coordinating center. At the end of the study, study data will be stored according to CSP guidelines and procedures. Retention of data will be conducted according to CSP operating procedures and federal and local VA regulations. This file will be destroyed according to CSP policy well after the close of the study.

Access to the study data is restricted to individuals with CSP approval. Study team members must be properly credentialed research staff and must be compliant with VA security trainings (e.g. HIPAA, Rules of Behavior, and Good Clinical Practices). Once formal training is completed, user accounts for a study-specific web application utilizing a URL specific to the study to access and use the system and enter patient data will be activated for project management, and study nursing staff.. Accounts will be password protected and unique to each user. The account permissions will correspond with the users' functional study group. Furthermore, the permissions of the electronic systems are heavily restricted. Only properly approved Coordinating Center personnel will have the ability to copy and export data. These individuals have received training on the local standard operating procedure (SOP) governing their permissions. Access to protected health information (PHI) will be restricted to individuals approved by CSP to have access to the data.

At the Boston CSPCC the following staff will have access to all forms of PHI:

1. Center Director
2. Study Director
3. Project Management
4. Study Nurses
5. Data Management
6. Biostatisticians
7. Quality Assurance Officer

8. SAS/Database Programmer
9. Research Assistant
10. Clinical Applications Coordinator
11. Informatics Team

Periodic access control assessments will be made by Coordinating Center Quality Assurance personnel to verify that access is controlled and appropriate for personnel. In addition, the CSPCC QA group will provide continuing education on good clinical practices compliance and will evaluate clinical site operations for violations of VA policies including VA data security policies and GCP.

At the end of the study, the data for this trial will remain property of the investigators and be stored and shared according to CSP guidelines and procedures. Retention and destruction of data will be conducted according to CSP operating procedures and federal and local VA regulations. This will include electronic data stored at the MAVERIC CSPCC, and at the VA facility housing our servers. Identifiable data will be kept according to CSP policy as outlined in the "CSP Investigator Guidelines for the Planning and Conduct of Cooperative Studies".

e. Data Management and Access Plan

Upon final analysis of the stated objectives in this protocol, the study plans to submit results for publication in scientific peer-reviewed journals and provide summary results on ClinicalTrials.gov. Results will be posted on ClinicalTrials.gov no later than one year after the last patient has been followed for 6 months after hospital discharge. After acceptance of the primary and other stated analyses by a journal, the Boston CSPCC will make these publication(s) available via the National Library of Medicine's PubMed Central within one year of the date of publication.

Digital data underlying primary scientific publications from this study will be held as part of a data sharing resource maintained by the CSPCC. Study data held for this purpose may include data, data content, format, and organization. The data may contain but are not limited to individually identifiable information, other PHI, and study codes. The data may be available to the public and other VA and non-VA researchers under certain conditions and consistent with the informed consent and CSP policy that prioritize protecting participants' privacy and confidentiality to the fullest extent possible. A detailed plan for data sharing will be developed in accordance with current technology, infrastructure, best practices, and policies and procedures in place at the time of oversight committee reviews (e.g., Privacy Board, Information Security, and Information Technology standards). The plan will include how data will be discovered, retrieved, analyzed, and managed and will note the materials that are available in machine-readable formats. This plan may be revised to ensure consistency with VA, including CSP, policies and standards for overall data sharing.

9. Statistical Analysis Plan

The proposed clinical trial is a prospective, randomized, controlled study. A total of up to 120 subjects will be randomized, stratified by study center, using response adaptive randomization based on the posterior probability of the active treatment (Treatment A) being superior to the standard of care (Treatment C) treatment.

The trial is designed to assess the superiority of treatment A compared to standard of care (treatment C) with respect to the primary endpoint, a composite endpoint of intubation or death during the hospitalization due to SARS-CoV-2 infection.

A Bayesian approach using a Beta-Binomial conjugate model is employed to (1) formally assess the superiority of Treatment A to Treatment C, and (2) to adaptively change the randomization ratio to favor the treatment arm favored by the data collected data.

This protocol is a major revision of an initial protocol that compared a dose of 200 mg of Sarilumab as the active treatment to standard of case. Release of preliminary interim results of an external study led to a change in dose from 200 mg to 400 mg. Before the change 9 subjects had been enrolled. Since no other major changes in the I/E criteria were made, the data of these 9 subjects enrolled under the previous protocol will be used in the analyses of the results for this study. It is our determination that this represents a conservative approach, because, when compared with SOC, the relative effect with 400 mg dose is expected to be at least as large as the one with 200 mg.

a. Study Objectives

The objective of this study is to assess the safety and efficacy of the active treatment in the treatment of subjects with moderate COVID-19 disease as defined by positive testing for SARS-CoV-2

b. Primary Endpoint

The primary endpoint is a composite endpoint of intubation or death within 14 days of enrollment, during hospitalization due to SARS-CoV-2 infection. To facilitate the adaptive approach described below, the primary endpoint will be assessed 7 days after approximately the 30th patient has been enrolled and approximately every additional 15th patient thereafter. The endpoint at day 7 is expected to be highly correlated with the endpoint at day 14, and data regarding that assumption will be collected.

We propose a standard Bayesian approach to calculate the current (posterior) probability that the primary endpoint event rate with treatment A is lower than the primary event rate with treatment C, given the accruing data so far. We assume that in each treatment group, the presence of the primary event outcome follows a Bernoulli distribution.

c. Random Adaptive Assignment of Patients

Patients are assigned to one of two treatment arms using an adaptive randomized scheme. Initially, the randomization will be balanced, then as data are accrued, assignment probabilities will be shifted in favor of arm that is performing better.

The first batch of subjects will be assigned using equal allocation (1:1) to both study arms (A = Active and C = SOC). Then, at each interim evaluation, the posterior probability, π, that the Event Rate in Group A is smaller than the Event Rate in Group C will be computed

$$\pi = P(\text{Event Rate in Group } A < \text{Event Rate in Group } C | \text{Data}),$$

where Data represents all cumulative outcome data available at the interim evaluation.
Thereafter, the value of π will determine the random assignments to treatment. In particular,

$$P(\text{next subject assigned to } A) \propto \pi^{\eta} \text{ and } P(\text{next subject assigned to } B) \propto 1-\pi^{\eta}.$$

where $\eta = 0.5$, is a calibration parameter.

The update for the value of π will occur every time the dataset of outcomes is updated, but for logistical simplicity the update will happen approximately after every 15 subjects enrolled.

d. Stopping Rule

We set two parameters δ_U and δ_L that govern how to stop the experiment either for overwhelming superiority or overwhelming futility, using the rules:

- STOP for superiority if: $\pi > \delta_U$
- STOP for futility if:

$$\pi_1 = P(\text{Event Rate in Group } A < \text{ Event Rate in Group } C + 3\% | \text{Data})$$

satisfy

$$\pi_1 < 1 - \delta_L$$

These two parameters are chosen so that the Type I error is <10%. We propose to calculate and set a maximum value for the total accrual, based on an extensive simulation study (see section f).

e. Statistical Methods

The null hypothesis is that the Active treatment will have a primary endpoint rate equal to or exceeding that of the SOC group.

The alternative hypothesis is that the Active treatment will have primary endpoint rate less than the SOC group rate. Specifically:

$$\text{H}_0: \pi_A - \pi_C \geq 0$$

$$\text{H}_\text{A}: \pi_A - \pi_C < 0$$

Where π_A is the true primary event rate for the Active treatment, π_C is the true primary event rate for the SOC arm.

Statistical analyses for the primary endpoint will be conducted using a statistical test for superiority of proportions based on posterior probability. Within each treatment group, a Beta (3, 12) prior will be

assumed for the true primary endpoint rate, π_A and π_C. This prior was selected to have a mean of 20% which is the average of the assumed rates under the alternative hypothesis and express *a priori* skepticism of the alternative hypothesis being true; indeed, under this prior, the prior probability of the alternative hypothesis is 50%, while the probability of observing an improvement in the true rate of 20% (the hypothesized improvement) is only 7.8%. **Thus, a reversal in this prior skeptical stance could only be due to strong support for the Active treatment in the data collected during the study.** With this choice regarding the prior distribution, in each group, the posterior probability distribution of the primary event rates will be Beta (3+# of events,12+#non-events). The criterion for success is based on the posterior probability of the alternative hypothesis (i.e., of superiority being met). The Active group will be declared superior to the SOC group if the posterior probability of the alternative hypothesis $\mathrm{H_A}$ is large, that is

$$P(H_1|Data) = P(\pi_A - \pi_C < 0|\text{Data}) > \delta_U.$$

Similarly, the Active group will be declared inferior to the SOC group if the posterior probability

$$P(\pi_A - \pi_C < 3\%|\text{Data}) < 1 - \delta_L.$$

The thresholds, δ_U and δ_L are a pre-specified level of evidence we require to declare the alternative hypothesis true. The thresholds will be selected such that the overall type I rate for testing the primary hypothesis of superiority is bounded by 10%.

A balanced randomization will be employed for approximately the first 30 subjects, after which the randomization probability will be assessed. A maximum of 6 interim looks will be scheduled after approximately each 15 enrolled subjects enter the study and have finished follow-up. At each interim analysis, the alternative hypothesis of superiority and the null hypothesis of inferiority will be evaluated. A final analysis will be conducted if no decision has been reached at the interim evaluations after 120 subjects have been enrolled.

f. Sample Size Determination

The assumptions for the calculations were elicited assuming a treatment dose of 200mg. It is our determination that this represents a conservative approach, because, when compared with SOC, the relative effect with 400 mg dose is expected to be at least as large as the one with 200 mg.
The assumptions for this analysis are:

- True primary event rate in the Active treatment group, π_A is 10.0%
- True primary event rate in the SOC treatment group, π_C is 30.0%
- Power 85%.
- Six (6) interim analyses and a final analysis will be conducted to evaluate both the superiority and futility
- Superiority assessment will be assessed using the posterior probability of the alternative hypothesis as specified above, where $\delta_U = 0.95$
- Futility assessment will be assessed using the posterior probability of the alternative hypothesis as specified above, where $\delta_L = 0.95$
- Randomization will be carried out in an adaptive Bayesian fashion. The randomization ratio will be evaluated at each of the 6 interim analyses

Rejection of the null hypothesis will signify that the Active is superior to the SOC with regards to primary endpoint event rates. A maximum total of 120 subjects, adaptively assigned to treatment A or C will have 85.7% power to reject the above null hypothesis in favor of the alternative under the stated assumptions. Since we are using the adaptive assignment, the group sizes are a function of the data and therefore random. Under the alternative hypothesis, it is expected that at the time when the decision is made, the median group size will be 42 in treatment A and 27 in treatment C; thus, if treatment A is superior to treatment C, more subjects will be treated with the more efficacious treatment. The one-sided type I error estimates are 9.4%. Additional operating characteristics of this design are presented in the shaded column of Table 1 (under the alternative hypothesis) and shaded column of Table 2 (under the null hypothesis). In the same tables, the operating characteristics of the proposed design are presented under additional scenarios (Scenarios A1 – A3). From the table, it can be seen that the power with the proposed method is sufficient under alternative scenarios.

Table 1: Operating characteristics of the Proposed Design (Alternative Hypothesis); *Group A* – Active Treatment, *Group C* – Standard of Care

	**Primary Scenario**	**Scenario A1**	**Scenario A2**	**Scenario A3**
True rate (Group A)	10.00%	5.00%	5.00%	5.00%
True rate (Group C)	30.00%	30.00%	25.00%	20.00%
Exit Probability (Group A)	85.7%	98.1%	93.4%	80.9%
Exit Probability (Group C)	0.0%	0.0%	0.0%	0.0%
Final Analysis (No Decision)	14.3%	1.9%	5.0%	81.0%
Mean Sample Size	74	57.3	68.0	82.1
Median Group A Size	42	30	38	49
Median Group C Size	27	21	24	30

Table 2: Operating characteristics of the Proposed Design (Null Hypothesis); *Group A* – Active Treatment, *Group C* – Standard of Care

	**Primary Scenario**
True rate (Group A)	30.00%
True rate (Group C)	30.00%
Exit Probability (Group A)	7.1%
Exit Probability (Group C)	2.1%
Final Analysis (No Decision)	90.6%

Mean Sample Size	115.3
Median Group A Size	58
Median Group C Size	60

10. Ethical Issues

a. Risks

The major risks to participation in this study include 1) immunosuppression, 2) possibly bowel perforation, and 3) adverse reaction to the medication. Medications that cause immunosuppression make patients more susceptible to certain kinds of infections, particularly bacterial infection. Although risks associated with sarilumab have only been observed in conditions in which the drug is given repeatedly (usually every 2 weeks) for a chronic disease, it is possible that one dose of sarilumab, which in this study twice as high (400 mg) as the dose that is used chronically (200 mg), might lead to a severe bacterial infection, necessitating treatment with antibiotics, treatment in the intensive care unit, or death. It is also possible that sarilumab might make SARS-CoV-2 infection worse, which could result in worsening respiratory failure, admission to the intensive care unit, or death.

Sarilumab is associated with a small increase in the risk of bowel perforation. This risk has only been observed so far in conditions in which the drug is given at the 200 mg dose repeatedly.

As of this version of the protocol, no study of the use of IL-6 inhibition for COVID-19 has reported an increase in the risk of bacterial infection following administration of anti-IL-6-R therapy, and no studies have reported bowel perforations in patients with COVID-19 pneumonia.

Individual participating sites and their respective IRB/R&D/Research Committee will decide whether this information warrants changes in the section on risk in the informed consent form.

As with any medication, there is the potential for an allergic reaction or an injection site reaction, although based on previous data both are rare with sarilumab. The subcutaneous injection may also cause discomfort.

In addition to the risks listed above, there may be previously unknown risks or side effects.

b. Potential Benefits

Because SARS-Co-V-2 emerged very recently, there is very limited data to guide clinical decision making. Anecdotal data strongly suggest that clinical decompensation is driven by a cytokine storm; thus, it is possible that early intervention with an IL-6 inhibitor might prevent progression to severe SARS-CoV-2. However, these medications also have side effects, including immunosuppression, that could worsen outcomes. The study is designed as a pragmatic, adaptive clinical trial with a play-the-winner design, which means that as time goes on, participants are more likely to receive the

treatment that is most likely to benefit them clinically. This design is chosen because it 1) provides rapid results, and 2) ensures that the highest proportion of patients benefit from participation in the clinical trial.

c. Analysis of Risks in Relation to Benefits

We currently do not know the best way to treat SARS-CoV-2 infection, although there is mounting evidence that the inflammatory response to the disease plays a major role in disease progression. This trial will help to answer if IL-6 inhibition, started early in the course of disease, can prevent progression and improve outcomes when added to standard of care. The randomization procedure includes a play-the-winner design, which gradually increases the number of patients who receive the treatment arm that yields the most benefit. Thus, the study is designed to 1) provide rapid results and 2) to optimize management. Given all this potential, we believe there is sufficient clinical equipoise to conduct this trial.

d. Stopping Rules

A participant may always withdraw their participation at any time.

- The study has stopping rules, based on futility; see Section 9, Statistical Analysis Plan
- A participant will be withdrawn from the study if the treating physician or investigator feels it is in the participant's best interest.

11. Safety Monitoring Plan

Good Clinical Practices and Site Review Program

a. Role of Good Clinical Practice

This trial will be conducted in compliance with the currently approved protocol, with the standards from the International Conference on Harmonisation Efficacy Guideline 6: Good Clinical Practice (ICH E6), and with applicable regulatory requirements. The intent of these regulations is to ensure the rights and well-being of trial participants are protected, and reported trial data are accurate, complete, and verifiable.

b. Clinical Monitoring

The Boston CSPCC Quality Assurance Team will monitor and assist investigators in complying with the above applicable regulations, GCP requirements, and VA policies. Study staff will receive GCP orientation at a study kickoff meeting (and/or subsequent meetings), or via computerized educational modules. QA Team members will provide training and materials to assist study personnel in organizing study files and will be available throughout the trial to advise and assist investigators regarding GCP issues.

Study site monitoring will be a combination of site visits, remote (off-site) monitoring performed by Boston CSPCC Quality Assurance staff, and centralized statistical monitoring. These visits and activities will be conducted according to the study's risk-based/integrated monitoring plan that will include ongoing periodic review of selected site performance indicator(s) as derived from the data submitted by

the site, findings identified during onsite or remote visits, and interaction with the local study site staff. All monitoring feedback will be reviewed periodically and the monitoring plan will be assessed for adequacy and effectiveness at least annually. The monitoring plan will be revised as necessary to reflect identified protocol-specific risks.

c. Quality Control through the Boston CSPCC Quality Management System

The Quality Management System (QMS) in place at the CSPCC will ensure further quality control for the trial. The Quality Assurance Department of the CSPCC will subject the data to risk-based audits and monitoring that will verify and validate data elements according to internal SOPs. In brief, a sample of the data will be verified at routine intervals. If errors are identified they will be referred to the data management team for resolution. If the error level rises to a predefined threshold, then the entire record or data element type will be subjected to verification and validation. Further, the CSPCC will conduct internal audits to ensure the quality of clinical trial processes and procedures. If deviations or non-conformances are identified they will be remedied through the internal corrective action/preventive action system of the QMS.

Risk based monitoring and auditing will also allow for the early systematic identification of problems that require remediation at the local site level. If problems are identified, the CSPCC will work with the study staff to create a remediation plan to address the issues. If problems persist after remediation, the site may be recommended for termination to the EC.

Study Organization and Administration

a. Administration

The administrative structure of this study is similar to others in the ‘CSP model’ and includes:

The CSPCC and the Study Chairs’ offices jointly will perform day-to-day scientific and administrative coordination of the study. These activities include developing and revising the study operational protocol, ICFs, and CRFs, ensuring the appropriate support for the participating center(s), scheduling meetings and conference calls, answering questions about the protocol, and conducting site visits. The CSPCC will also prepare interim and final progress reports, reports on metrics, and archive study data at the end of the study. Archiving study records per VHA Handbook 1200.05 requires all study records be retained until disposition instructions are approved by the National Archives and Records Administration and are published in VHA‘s Records Control Schedule (RCS 10-1). Study DMC reports will be produced approximately every 6 months (exact frequency dependent on DMC determination) and other reports will be produced at intervals determined by the study team. Participant accrual, participant safety, and data quality will be monitored closely by the CSPCC at least annually (or at an interval determined by the DMC) to ensure the study is progressing satisfactorily. Further delineation of responsibilities will be documented in communications with the Study Chairs’ and CSPCC offices.

b. Monitoring

The following groups monitor various aspects of the study. These committees will meet according to current CSP guidelines as a model.

The Executive Committee (EC) is responsible for oversight of the operations of the study, including protocol amendments, and overall management of the study. It will be headed by the Study Chairs and Study Directors and will include the study Biostatistician, CSPCC Center Director, selected participating investigators, and outside consultants as needed. This committee will meet regularly to review data and may take the following actions: recommend changes to the study, help determine outcomes for sites whose performance is substandard, initiate any sub-protocols, and discuss publication of the study results. This Committee must grant permission before any study data may be used for presentation or publication.

The Data Monitoring Committee (DMC) will review the progress of the study and monitor participant intake, outcomes, AEs, and other issues related to participant safety. Interim, independent, and unbiased reviews of study progress will be provided. The DMC consists of experts in the study's subject matter field(s), clinical trials, biostatistics, and ethics. These individuals will not be participants in the trial and will not have participated in the planning of the protocol. The DMC will consider safety or other circumstances as grounds for early termination, including compelling internal or external evidence of treatment differences or the unfeasibility of addressing the study hypothesis (e.g., poor participant intake, poor adherence to the protocol). The DMC will monitor the primary endpoint at pre-determined intervals and recommend whether the trial should be stopped for efficacy.

At each of its meetings during the study period, the DMC will review randomization rates and assess the difference between actual and projected rates, as well as the impact of these assessments on overall sample size. If the study enrollment is inadequate, the reasons for exclusion may be scrutinized and actions may be suggested. An assessment of whether the trial should continue will be made followed by recommendations, as appropriate. All SAEs will be reported regularly at these meetings for DMC review. The study Biostatistician will provide the appropriate data to the DMC at specified intervals for this purpose. Conditional power estimates may be provided at their request to assist the DMC in making decisions and recommendations. To help them make assessments, the Study Chairs, Study Directors and the study Biostatistician will furnish the DMC with appropriate monitoring data before each meeting. The DMC makes recommendations after each meeting to the CSP Director about whether the study should continue or be stopped.

The VA Boston Institutional Review Board will monitor the study's SAEs on a continual basis. They may conduct annual reviews of the study.

The CSPCC Human Rights Committee (HRC) is a diverse committee composed primarily of individuals from varying demographic and professional backgrounds and includes a number of Veteran members. The HRC is responsible for ensuring participant's rights and safety are upheld prior to study initiation and during the conduct of the study. The committee reviews all new protocols, informed consent documents, select protocol amendments, periodically makes site visits to participating centers to monitor firsthand the progress of the study and may be asked to review any ethical and human rights issues that arise during the conduct of the study.

c. Safety Monitoring Plan

Monitoring side effects and adverse events in the traditional manner of usual clinical trials is not feasible for this clinical trial. First, this is an embedded pragmatic Point of Care study in which all data is captured actively or passively through the EHR, and thus real-time reporting is not possible, except for deaths that occur during hospitalization for COVID-19. Second, during the COVID-19 national pandemic we need to reduce the exposure of study staff to infected patients. The only study-related burden bringing additional risk of exposure to clinical staff is the need for the injections of sarilumab, which is similar in nature to other aspects of standard clinical care. Finally, real-time monitoring will not assure adequate (or timely) safety of human subjects beyond that already done by the medical staff as part of routine medical care on this acutely ill inpatient population. Accordingly, we propose an alternative safety reporting plan for this study that ensures protection of the participants and that complies with VHA Policy (i.e., VHA Directive 1200.05 and VHA Handbook 1058.01).

The participant's physicians, nurses, and other health providers will continue their usual monitoring of the subject throughout the course of his/her treatment. If any treatments are indicated, they will be provided by health providers as a part of the participant's routine medical care. The CSPCC staff will collect safety data from the medical record from the time of consent through the end of the study period. The safety data will then be aggregated and classified according to ICD-10 codes.

d. Safety Reporting

Aggregated safety data will be reported to the Data Monitoring Committee, the study Executive Committee, and the VA IRB.

Definitions and recording requirements for this trial are based on the International Conference on Harmonisation Efficacy Guideline 2a: Pharmacovigilance: Clinical Safety Data Management: Definitions and Standards for Expedited Reporting (ICH-E2A), the Code of Federal Regulations (21 CFR 312.32) and CSP Global Standard Operation Procedure (SOP) 3.6.

Given the comorbidity expected in this acutely ill study population, it is anticipated that a large number of AEs will be observed, most of which will not be related to study drug. For this reason, the study will collect reports of all SAEs, but only those non-serious AEs that, in an investigator's clinical judgment, are at least possibly attributed to a study drug and cannot be attributed to non-study intervention causes (e.g., to comorbid conditions, the disease that is the subject of the study, or non-study drugs). A list of expected AEs is included below.

1. Adverse Events

Adverse events will be collected using the 21 CFR 312.32, International Conference on Harmonisation (ICH) for Clinical Safety Data Management (ICH-E2A), and CSP Global SOP 3.6 definitions. Adverse events (AEs) are defined by the 21 CFR 312.32 as "any untoward medical occurrence associated with the use of a drug in humans, whether or not considered drug related." According to ICH-E2A, "an AE, therefore, can be any unfavorable or unintended sign (including an abnormal laboratory finding), symptom, or disease temporally associated with the study interventions."

Expected adverse events of interest will be culled from the EHR as part of the outcome ascertainment activities of this protocol. The expected AEs of interest as identified in this trial are:

- Neutropenia: neutrophil count <1000 mcL
- Thrombocytopenia (low platelet count): <75,000 mcL
- Non-COVID related bacterial, fungal, or mycobacterial infection
- Oral herpesvirus infection, herpes zoster infection, cytomegalovirus infection
- ALT >3-fold increase above the upper limit normal (ULN)
- Bowel perforation

These safety events are monitored and treated as part of routine medical care. These events and outcomes will be identified by the CSPCC through the EHR. For the first 30 days after randomization CSPCC staff will review the EHR daily or every one to two days for the first 14 days and then weekly for the remainder of the 30-day period to assess for potential effects of the study drug. Data will be entered on an Access form and uploaded to a secure server space. Beyond the 30 days informatics staff at the Boston CSPCC will extract medical record data on all subjects and identify adverse events through ICD-10 codes, laboratory values, and medication files. Expected adverse events will be reported to the DMC in semi-annual reports. The DMC also reserves the right to request reporting on additional safety data not specified above, as relevant to the study. Data will be in the form of aggregated data tables detailing the frequencies of these events by blinded treatment group. These events will be reported to the IRB in blinded aggregate form at the time of continuing review.

Adverse events which develop into Serious Adverse Events, as defined below, will be reported as such.

2. Expected Serious Adverse Events

Serious adverse events (SAEs) are a subset of adverse events defined in 21 CFR 312.32(a) and VA Handbook 1058.01 paragraph 4(w), as follows:

Definition of SAE from CFR 312.32 (a): Serious adverse event. An adverse event or suspected adverse reaction is considered "serious" if, in the view of either the investigator or sponsor, it results in any of the following outcomes: death, a life-threatening adverse event, inpatient hospitalization or prolongation of existing hospitalization, a persistent or significant incapacity or substantial disruption of the ability to conduct normal life functions, or a congenital anomaly/birth defect. Important medical events that may not result in death, be life-threatening, or require hospitalization may be considered serious when, based upon appropriate medical judgment, they may jeopardize the patient or subject and may require medical or surgical intervention to prevent one of the outcomes listed in this definition.

Definition of SAE according to VA Handbook 1058.01: An SAE is an AE in human research that results in death, a life-threatening experience, inpatient hospitalization, prolongation of hospitalization, persistent or significant disability or incapacity, congenital anomaly, or birth defect. An AE is also considered serious when medical, surgical, behavioral, social, or other intervention is needed to prevent such an outcome.

This study will not use MedDRA coding of AE and SAE data. The study team will define events using the data sources described above and categorize events by system organ class, major diagnostic category, or assessment type for reporting.

Note: Intubation is a study outcome but may also be reported as an SAE depending on local IRB requirements. Death is also a study outcome but could be a result of COVID-19 infection or a result of another process. All deaths will be reported to the IRB on the same timeline as SAEs, with description to allow determination whether they should be considered purely as study outcomes (progression of COVID-19 to respiratory failure or multi-organ-system failure) or also as treatment-emergent SAEs.

3. Unanticipated Problems Involving Risks to Subjects and Others

Any unanticipated problems involving risks to subjects or others (UPRs), but not qualifying as a serious adverse event by definition (such as errant distribution of study medication), will also be reported. Unanticipated problems related to the study design will be reported to the IRB in an expedited fashion per IRB policies (within 5 business days of identifying the problem). Examples of possible events include failure to distribute study drug or serious adverse drug reactions. Informatics staff at the Boston CSPCC will extract medical record data on all subjects regularly allowing for the identification of UPRs at fixed intervals. Study reports will be circulated to appropriate members, including the study chair.

12. Adverse Event/Unanticipated Problems Reporting Plans

The Principal Investigator will report Unanticipated Problems, Adverse Events, and safety monitors' reports to the IRB in accordance with VHA Handbook 1058.01 and VABHS IRB SOP.

All serious adverse events as defined above that are feasibly identified through the medical record will be reported to the VHABHS IRB as they occur and in aggregate form per the timelines specified by the IRB. SAEs will also be reported in aggregate to the DMC at 6-month intervals. Study reports will also be circulated to appropriate study team members, including the study investigators and the DMC.

UPRs that are related to the research, including those that result in an SAE, pursuant to the definition above, will be reported to the VA Boston IRB within 5 business days by the Boston CSP Coordinating Center after becoming aware of the event.

13. References

1. Chen Z, et al. Efficacy of hydroxychloroquine in patients with COVID-19: results of a randomized controlled trial. 2020 [online pre-print].
2. Beigel J, et al. Remdesivir for the treatment of Covid-19 – Final report. N Engl J Med 2020 Nov 5;383(19):1813-1826. doi: 10.1056/NEJMoa2007764. Epub 2020 Oct 8.
3. Chen G, et al. Clinical and immunologic features in severe and moderate coronavirus disease 2019. J Clin Invest 2020 [online ahead of print].
4. Deng Y, et al. Clinical characteristics of fatal and recovered cases of coronavirus disease 2019 (COVID-19) in Wuhan, China: a retrospective study. Chin Med J 2020 [online ahead of print].

5. To KKW, et al. Temporal profiles of viral load in posterior oropharyngeal saliva samples and serum antibody responses during infection with SARS-CoV-2: an observational cohort study. Lancet Infect Dis 2020 [online ahead of print].
6. Xu X, et al. Effective Treatment of Severe COVID-19 Patients with Tocilizumab. 2020 [online pre-print, http://chinaxiv.org/abs/202003.00026].
