# Supplementary material for: Subcutaneous sarilumab for the treatment of hospitalized patients with moderate to severe COVID19 disease: A pragmatic, embedded randomized clinical trial
Source: PLoS One. 2022 Feb 25;17(2):e0263591. doi: 10.1371/journal.pone.0263591 (PMC8880885; doi:10.1371/journal.pone.0263591)
Supplement: Attachment — Submitted filename: Response to Reviewers.pdf [file pone.0263591.s010.pdf]

VA | U.S. Department of Veterans Affairs

Westyn Branch-Elliman, MD, MMSc
Associate Professor of Medicine
VA Boston Healthcare System
Medical Service* (111)*, Infectious Diseases Section
1400 VFW Parkway
West Roxbury, MA 02132-4927

BOSTON UNIVERSITY

Giuseppe Vittorio De Socio, MD, PhD
Academic Editor, *PLOSOne*

November 18, 2021

Dear Dr. De Socio:

Thank you very much for the opportunity to revise and improve our manuscript "*Subcutaneous Sarilumab for the Treatment of Hospitalized patients with Moderate to Severe COVID19 Disease: A Pragmatic, Embedded Randomized Clinical Trial.*" Based on editorial and reviewer feedback, we have edited our manuscript and hope that it is now suitable for publication in *PLOSOne.* A detailed response to comments is included at the end of this letter.

All authors contributed significantly to the work presented and reviewed and approved the revised manuscript prior to submission. The manuscript has not been previously published and is not being considered for publication elsewhere. Grant support and potential conflicts of interest are listed at the end of the document. WBE, PM, and JMS were site investigators for a study funded by Gilead Sciences (funds to institution). All other authors have no conflicts of interest to report. The funders had no role in the design, data collection and analysis, decision to publish, or preparation of the manuscript. Per national VA policy and the patient consent form, deidentified data will be made available to qualified investigators through a Data Use Agreement approved by the institution's Information Security Officer, Privacy Officer, and Research and Development Committee.

Corresponding author:
Westyn Branch-Elliman, MD, MMSc
1400 VFW Parkway
West Roxbury, MA 02132
Westyn.branch-elliman@va.gov/wbranche@bidmc.harvard.edu

Thank you very much in advance for your time and consideration. Please do not hesitate to contact me with any questions regarding this submission.

Sincerely,

Westyn Branch-Elliman, MD, MMSc, FSHEA

When submitting your revision, we need you to address these additional requirements.

1. Please ensure that your manuscript meets PLOS ONE's style requirements, including those for file naming. The PLOS ONE style templates can be found at https://journals.plos.org/plosone/s/file?id=wjVg/PLOSOne_formatting_sample_main_body.pdf and https://journals.plos.org/plosone/s/file?id=ba62/PLOSOne_formatting_sample_title_authors_affiliations.pdf

   Response: These recommendations have been reviewed and the manuscript is in compliance with journal style recommendations.

2. Please provide additional details regarding participant consent. In the ethics statement in the Methods and online submission information, please ensure that you have specified what type you obtained (for instance, written or verbal, and if verbal, how it was documented and witnessed).

> **Response**: We updated the section to clarify informed consent processes, and added a reference to a paper published by our group about the processes used for this study. The section now reads, "After clarifications to the initial US Food and Drug Administration (FDA) guidance were issued,[22] written documentation of informed consent was obtained from all participants or their legally authorized representative. Details of the informed consent processes, which were developed to minimize research staff exposure and also limit the use of personal protective equipment at a time when resources were scarce, are previously published.[23]"

3. Thank you for submitting your clinical trial to PLOS ONE and for providing the name of the registry and the registration number. The information in the registry entry suggests that your trial was registered after patient recruitment began. PLOS ONE strongly encourages authors to register all trials before recruiting the first participant in a study.

As per the journal's editorial policy, please include in the Methods section of your paper:
1) your reasons for your delay in registering this study (after enrolment of participants started);
2) confirmation that all related trials are registered by stating: "The authors confirm that all ongoing and related trials for this drug/intervention are registered".

> **Response**: We have added text addressing item 1 and have added the text specified in item 2. The study was designed and implemented in 10 days under circumstances of medical urgency in the hospital and no data to guide management of patients with moderate-to-severe Covid-19, so we elected to begin enrollment prior to completing registration in clinicaltrials.gov. This is clarified in the revision. The authors confirm that all ongoing and related trials are registered, and the requested language has been added in the text.

4. Thank you for stating the following financial disclosure:
[WBE was supported by NIH NHLBI 1K12HL138049-01.
This material is the result of work supported with resources and the use of facilities the VISN-1 Clinical Trials Network and the VA Boston Healthcare System.]

Please state what role the funders took in the study. If the funders had no role, please state: "The funders had no role in study design, data collection and analysis, decision to publish, or preparation of the manuscript."
If this statement is not correct you must amend it as needed.
Please include this amended Role of Funder statement in your cover letter; we will change the online submission form on your behalf.

> **Response**: There was no role of the funder in the design or implementation of the study. We have added this sentence in the section on Funding and in the cover letter.

5. Thank you for stating the following in the Competing Interests section:
[WBE, PM, and JMS were site investigators for a study funded by Gilead Sciences (funds to institution). All other authors have no conflicts of interest to report.]

Please confirm that this does not alter your adherence to all PLOS ONE policies on sharing data and materials, by including the following statement: "This does not alter our adherence to PLOS ONE policies on sharing data and materials." (as detailed online in our guide for authors http://journals.plos.org/plosone/s/competing-interests). If there are restrictions on sharing of data and/or materials, please state these. Please note that we cannot proceed with consideration of your article until this information has been declared.

Please include an updated competing interests statement in your cover letter; we will change the online submission form on your behalf.

> **Response**: We have added the requested sentence, and additional language related to VA regulations regarding sharing of data only through approved Data Use Agreements as part of a data sharing statement. We have also included an updated competing interests statement in the submission letter.

6. In your Data Availability statement, you have not specified where the minimal data set underlying the results described in your manuscript can be found. PLOS defines a study's minimal data set as the underlying data used to reach the conclusions drawn in the manuscript and any additional data required to replicate the reported study findings in their entirety. All PLOS journals require that the minimal data set be made fully available. For more information about our data policy, please see http://journals.plos.org/plosone/s/data-availability.

Upon re-submitting your revised manuscript, please upload your study's minimal underlying data set as either Supporting Information files or to a stable, public repository and include the relevant URLs, DOIs, or accession numbers within your revised cover letter. For a list of acceptable repositories, please see http://journals.plos.org/plosone/s/data-availability#loc-recommended-repositories. Any potentially identifying patient information must be fully anonymized.

Important: If there are ethical or legal restrictions to sharing your data publicly, please explain these restrictions in detail. Please see our guidelines for more information on what we consider unacceptable restrictions to publicly sharing data: http://journals.plos.org/plosone/s/data-availability#loc-unacceptable-data-access-restrictions. Note that it is not acceptable for the authors to be the sole named individuals responsible for ensuring data access.

We will update your Data Availability statement to reflect the information you provide in your cover letter.

> **Response**: We updated the data availability statement to clarify VA policy, and added this information to the cover letter. Deidentified data will be made available to qualified investigators through a Data Use Agreement approved by the institution's Information Security Officer, Privacy Officer, and Research and Development Committee, per national VA policy and per what was included in the informed consent form.

[Note: HTML markup is below. Please do not edit.]

Reviewers' comments:

Reviewer's Responses to Questions

Comments to the Author

1. Is the manuscript technically sound, and do the data support the conclusions?

The manuscript must describe a technically sound piece of scientific research with data that supports the conclusions. Experiments must have been conducted rigorously, with appropriate controls, replication, and sample sizes. The conclusions must be drawn appropriately based on the data presented.

Reviewer #1: Yes

Reviewer #2: Yes

---

2. Has the statistical analysis been performed appropriately and rigorously?

Reviewer #1: Yes

Reviewer #2: Yes

---

3. Have the authors made all data underlying the findings in their manuscript fully available?

The PLOS Data policy requires authors to make all data underlying the findings described in their manuscript fully available without restriction, with rare exception (please refer to the Data Availability Statement in the manuscript PDF file). The data should be provided as part of the manuscript or its supporting information, or deposited to a public repository. For example, in addition to summary statistics, the data points behind means, medians and variance measures should be available. If there are restrictions on publicly sharing data—e.g. participant privacy or use of data from a third party—those must be specified.

Reviewer #1: Yes

Reviewer #2: No

> **Response**: Deidentified data will be made available to investigators through a data use agreement, per national VA policy and per what was included in the informed consent. This is clarified in the data availability statement and outlined in our cover letter.

---

4. Is the manuscript presented in an intelligible fashion and written in standard English?

PLOS ONE does not copyedit accepted manuscripts, so the language in submitted articles must be clear, correct, and unambiguous. Any typographical or grammatical errors should be corrected at revision, so please note any specific errors here.

Reviewer #1: Yes

Reviewer #2: Yes

---

5. Review Comments to the Author

Please use the space provided to explain your answers to the questions above. You may also include additional comments for the author, including concerns about dual publication, research ethics, or publication ethics. (Please upload your review as an attachment if it exceeds 20,000 characters)

Reviewer #1: This is an interesting study. Given the pandemic, the play-the-winner design was appropriate. There are a couple of concerns. One concern is the small sample size based on which the conclusion was reached. Second concern is the initial 200mg dose, and addition of remdesivir and dexamethasone to SOC. These made it difficult to interpret the findings.

> **Response**: We appreciate the reviewer's interest in the study and the justification for its design. We had attempted to fully acknowledge this scientific weakness in the original discussion:
>
> "The plan to have the trial "adapt" to changing circumstances and a changing SOC in the setting of a novel and life-threatening disease is both a strength (ethically) and a weakness (scientifically and operationally). In 2 of 10 total amendments, a significant inclusion criterion (degree of hypoxemia) and the dose of study drug were changed, at the same time that underlying SOC changed, so the first 9 patients and the last 41 patients were treated differently. Other trials have changed outcome measures and limited exclusion criteria based on concomitant treatment, for similar and equally appropriate reasons."
>
> To address the reviewer comment, we added the following language: "Although the results limited to the period when the 400 mg dose was used, which was also a time when dexamethasone and remdesivir also became widely used, were not entirely convincing for lack of benefit on their own, the probability of benefit was only 11% based

> on an event rate of 2/15 in the sarilumab arm versus 0/25 in the control arm, which in the context of earlier results was sufficient to stop the trial."

Some clarifications are needed as in the specific comments.

Page 6, in section Study design:
"Per pre-specified plans for a play-the-winner design, the first 30 patients were randomized 1:1 to sarilumab or no additional treatment beyond the current SOC, as determined by treating physicians." Was the randomization determined by treating physicians or by a randomization scheme?

> **Response**: We agree that the language was confusing. The standard of care was determined by the treating physicians, not the randomization. The section now reads, "Per pre-specified plans for a play-the-winner design, the first 30 patients were randomized 1:1 to sarilumab or no additional treatment beyond the current SOC. SOC was determined by the treating physicians and local treatment guidance and not pre-determined by investigators."

"Following a pre-specified scheme, the study statistician determined the change in randomizationbased on the observed results." Please add details on the randomization scheme so that it is clear to the readers.

> **Response**: We agree that more detail is needed for clarity. To address this issue, we now refer to the study protocol, which are included as supplemental documents. Details of the adaptive randomization process are outlined in these documents.

"Neither the allocation ratio nor randomization list was shared with the study team. Randomization was not stratified according to recruitment site. Investigators were blinded to the results and randomization ratio." What does it mean "blinded to the results"? A statistician would infer it mean the investigators were blinded on what treatment the patient was receiving. Please clarify. Did you use placebo to ensure the blinding? If no placebo was used, what's the reason for that decision? What measure was taken to ensure no bias was introduced when the study endpoint was assessed?

> **Response**: We apologize for the confusion caused by the way the statements were phrased. Neither the investigators not the patients were blinded to the intervention, but investigators were blinded to aggregate outcomes that determined the adaptive randomization, and also to the updated randomization ratio. Based on reviewer feedback, we have revised the text on page 7 to make the process more clear. We did not take any specific measure to ensure no bias was introduced into the assessment of outcomes; however, we chose only objective outcomes (mechanical ventilation or death) in order to limit the impact of this potential source of bias. Use of placebo would have dramatically increase the cost and regulatory complexity of the study.

Reviewer #2: This is a well written manuscript. I only have one concern.
The number of primary outcomes listed in Table 4 does not appear to match those listed in the abstract and main text of the Results section, for example, 5 (Table 4) versus 6 (text) in the Sarilumab group.

Response: We apologize for reporting the primary outcome differently in different places and have corrected the abstract so that the numbers refer to the outcome occurring within 14 days. The text on p. 11 has been corrected to provide numbers for the 14-day and 30-day outcomes, and deaths were adjudicated based on 30 days. The tables included both 14- and 30-day outcomes and are not updated.

6. PLOS authors have the option to publish the peer review history of their article (what does this mean?). If published, this will include your full peer review and any attached files.

If you choose "no", your identity will remain anonymous but your review may still be made public.

Do you want your identity to be public for this peer review? For information about this choice, including consent withdrawal, please see our Privacy Policy.

Reviewer #1: No

Reviewer #2: No

[NOTE: If reviewer comments were submitted as an attachment file, they will be attached to this email and accessible via the submission site. Please log into your account, locate the manuscript record, and check for the action link "View Attachments". If this link does not appear, there are no attachment files.]

While revising your submission, please upload your figure files to the Preflight Analysis and Conversion Engine (PACE) digital diagnostic tool, https://pacev2.apexcovantage.com/. PACE helps ensure that figures meet PLOS requirements. To use PACE, you must first register as a user. Registration is free. Then, login and navigate to the UPLOAD tab, where you will find detailed instructions on how to use the tool. If you encounter any issues or have any questions when using PACE, please email PLOS at figures@plos.org. Please note that Supporting Information files do not need this step

Response: The figure files have been reviewed and are compatible with the Plos system.
